# The role of noncoding mutations in blood cancers

**DOI:** 10.1242/dmm.041988

**Published:** 2019-11-26

**Authors:** Sunniyat Rahman, Marc R. Mansour

**Affiliations:** Department of Haematology, UCL Cancer Institute, University College London, London WC1E 6DD, UK

**Keywords:** Enhancers and promoters, Gene regulation, Haematological malignancy, Noncoding genome

## Abstract

The search for oncogenic mutations in haematological malignancies has largely focused on coding sequence variants. These variants have been critical in understanding these complex cancers in greater detail, ultimately leading to better disease monitoring, subtyping and prognostication. In contrast, the search for oncogenic variants in the noncoding genome has proven to be challenging given the vastness of the search space, the intrinsic difficulty in assessing the impact of variants that do not code for functional proteins, and our still primitive understanding of the function harboured by large parts of the noncoding genome. Recent studies have broken ground on this quest, identifying somatically acquired and recurrent mutations in the noncoding genome that activate the expression of proto-oncogenes. In this Review, we explore some of the best-characterised examples of noncoding mutations in haematological malignancies, and highlight how a significant majority of these variants impinge on gene regulation through the formation of aberrant enhancers and promoters. We delve into the challenges faced by those that embark on a search for noncoding driver mutations, and provide a framework distilled from studies that have successfully identified such variants to overcome some of the most salient hurdles. Finally, we discuss the current therapeutic strategies being explored to target the oncogenic mechanism supported by recurrent noncoding variants. We postulate that the continued discovery and functional characterisation of somatic variants in the noncoding genome will not only advance our understanding of haematological malignancies, but offer novel therapeutic avenues and provide important insights into transcriptional regulation on a broader scale.

## Abstract (precis)

**Summary:** This Review highlights the challenging but rewarding search for somatic mutations in the noncoding genome, and how such variants nucleate aberrant enhancers and promoters that drive the expression of proto-oncogenes.

## Introduction

Haematological malignancies cover a broad spectrum of cancers of bone marrow, lymphatic or thymic origin. These malignancies arise through the acquisition of genetic aberrations that drive proliferation, inhibit differentiation, enable cellular survival and evade immune surveillance. Haematological malignancies are the fourth most common cause of cancer mortality, surpassed only by lung, colorectal and breast cancer (www.cancerresearchuk.org). Huge strides have been made in treatment, with notable improvements in acute lymphoblastic leukaemia (ALL), which now has a survival rate of more than 90% for those aged 14 or younger (www.cancerresearchuk.org). Unfortunately, 5-year survival in adult acute myeloid leukaemia (AML) and ALL are still less than 50%, with significant treatment challenges including treatment-resistant disease, clonal heterogeneity of the underlying disease, treatment-associated toxicities and poor tolerance for intensive treatment regimens, particularly in older patients with comorbidities (Kansagra et al., 2018). Because of this, there is a significant impetus to discover new genetic aberrations, including driver mutations (see Box 1 for a glossary of terms), that may provide insight into the mechanisms of malignant haematopoiesis, as well as offering novel therapeutic opportunities.

**Box 1. Glossary**

**APOBEC:** ‘Apolipoprotein B mRNA editing enzyme, catalytic peptide-like’ are a family of catalytically active proteins that can insert mutations in both DNA and RNA through the deamination of cytidine to uridine, which is believed to be a significant endogenous mutagen in cancers.

**ATAC-seq:** ‘Assay for transposase-accessible chromatin using sequencing’ is a technique used to identify nucleosome-free regions of the genome. Such open regions of chromatin are susceptible to digestion by Tn5 transposase, which simultaneously digests and ligates adapter sequences for high throughput sequencing.

**Chimeric fusions:** A class of mutations that create a new protein by fusing two or more different coding sequences.

**Chromatin immunoprecipitation (ChIP):** An antibody-based experimental technique that identifies the location of protein binding events to DNA at nucleotide resolution. The immunoprecipitated DNA fragments can be sequenced (a technique called ChIP-seq) and mapped to the reference genome sequence.

**Chromatin interaction analysis by paired-end tag sequencing (ChIA-PET):** A technique used to detect interactions between disparate DNA sequences via a protein of interest. This is achieved by combining chromatin immunoprecipitation of a protein of interest, proximity ligation of DNA fragments and high throughput sequencing.

**Class switch recombination:** Allows B cells to rearrange the constant region genes in the immunoglobulin heavy chain locus to switch expression from one class of immunoglobulin to another.

**Cohesin:** A ring-shaped protein complex that is required for sister chromatid cohesion and to make contacts between distal chromatin segments for gene regulation.

**DNase hypersensitivity sequencing (DNase-seq):** A technique that allows for the sequencing of accessible genomic regions as determined by sensitivity to cleavage by DNase. These sites may have regulatory potential and are often bound by transcription factors and coactivators.

**Driver mutations:** A class of mutations that confer a growth or survival advantage in cancerous cells and thus promote cancer development.

**Enhancer:** A regulatory sequence element that can alter the transcriptional output of a gene.

**Hi-C:** A technique used to capture the conformational organisation of genomes. Briefly, it involves chromatin fixation, digestion and ligation, which also introduces a biotin-labelled nucleotide at ligation junctions. Following biotin-selective purification, the DNA fragments can be subjected to high-throughput sequencing to map many-to-many chromatin contacts and interactions.

**Indels:** Small nucleotide insertions or deletions or a combination of both at specific genomic loci.

**Insulator:** A regulatory sequence that creates a boundary between enhancers and promoters to minimise the activity of these sequences on gene regulatory processes.

**Kataegis:** A form of localised hypermutation that is characterised by clusters of C>T and/or C>G mutations that are substantially enriched at TpCpN trinucleotides and on the same DNA strand.

**Monoallelic expression:** Where expression of a gene is identified as being from a single allele only, either maternal or paternal.

**MonoMAC syndrome:** ‘Monocytopaenia and mycobacterial infection’ syndrome arises from heterozygous mutations in *GATA2* leading to loss of function. This gene is critical for functional haematopoiesis and lymphatic formation, so loss of function leads to significantly reduced numbers of circulating monocytes, dendritic cells, natural killer and B cells, as well as an increased likelihood of opportunistic infections and haematological malignancies.

**Promoter:** A regulatory sequence element nearest to the transcriptional start site of a gene that is bound by the core transcriptional machinery, including RNA pol II, and capable of activating gene expression.

**Transcription factor (TF):** A protein that binds specific DNA sequences through a DNA binding domain, and that can activate or repress gene expression.

**Transcription start site (TSS):** The nucleotide position of transcriptional initiation, which usually corresponds to the 5′ cap of an mRNA transcript.

**V(D)J recombination:** An endogenous mutagenic process that facilitates the recombination of V, D and J gene segments of developing T and B cells that results in diverse T cell receptor and immunoglobulin repertoires, respectively.

Detailed genetic characterisation of haematological malignancies has already identified alterations that are now being used for better diagnosis, prognostication, subtype identification and to inform therapeutic decisions (Taylor et al., 2017). The vast majority of these genetic alterations have been identified by studies focused on the coding sequences, which represent just 2% of the human genome, leaving the noncoding genome largely unexplored (ENCODE Project Consortium, 2012). Here, we discuss examples of noncoding mutations that have been identified in haematological malignancies so far, and explore how these examples have shaped our understanding about what constitutes a functional or driver noncoding mutation. Furthermore, we describe the challenges in identifying noncoding mutations that are drivers, rather than passengers, within the trajectory of cellular transformation, and begin to outline a framework through which one can potentially address some of these challenges to identify novel noncoding mutations that have functional significance. Finally, we provide some insight into therapeutic strategies that are currently being explored to disrupt the oncogenic mechanisms that arise from noncoding oncogenic mutations.

## Rationale for the identification and characterisation of mutations in the noncoding genome

There is a strong rationale for exploring the noncoding genome for biomarkers, therapeutic targets and somatically acquired driver mutations (Box 1). First, it has become clear that the noncoding genome itself is rich with *cis*-regulatory DNA elements such as promoters, enhancers and insulators (Box 1) (ENCODE Project Consortium, 2012). Whereas promoters bind the core transcriptional machinery, including RNA polymerase II, enhancers are able to bind regulatory proteins called transcription factors (TFs; Box 1) that can dramatically alter the activity of promoters, even from distal positions within the genome and without orientation constraints (Haberle and Stark, 2018). Ultimately, these regulatory mechanisms ensure that genes are expressed at an appropriate time and magnitude during the trajectory of cellular differentiation. This is especially true in haematopoiesis, where master TFs such as GATA1, SPI1, RUNX1 and MYB intimately regulate the expression of genes critical for the development of cells from erythroid, myeloid and lymphoid lineages (Jagannathan-Bogdan and Zon, 2013). Therefore, thorough interrogation of the noncoding genome is warranted in the context of haematological malignancies in which mutations disrupt nominal haematopoietic developmental programmes. Under these circumstances, one can hypothesise that novel uncharacterised variants may reside in the noncoding genome, particularly in regions with regulatory potential.

Secondly, the continued advancement of next generation sequencing (NGS) technologies has allowed for better exploration of cancer genomes. There are now several detailed and integrated datasets of human malignancies available thanks to large-scale collaborative efforts. These include The Cancer Genome Atlas (TCGA), which has generated data on 27 cancer types (including AML) through exome sequencing, copy number variation analysis using single nucleotide polymorphism (SNP) arrays, DNA methylation and RNA sequencing (Cancer Genome Atlas Research Network et al., 2013a,b). Additional data has been collated and generated by the Catalogue of Somatic Mutations In Cancer (COSMIC) project, which has reported approximately six million coding mutations and explored other genetic mechanisms that can spearhead cancer progression, including gene fusions, drug resistance mutations and, more recently, noncoding mutations (Tate et al., 2019). In the context of haematological malignancies, focused studies on specific diseases, such as T cell ALL (T-ALL) and AML, have identified coding variants, chromosomal translocations, chimeric fusions (Box 1) and mutations, which have then been coupled with clinical outcome data for improved prognostication (Liu et al., 2017; Papaemmanuil et al., 2016; Zhang et al., 2012). These studies have been essential in distilling important details about the pathogenesis of these malignancies and the mechanisms by which specific mutations disrupt normal cellular function, yet the vast majority of these studies have reported mutations only in the exome, leaving the noncoding genome relatively unexplored.

Thirdly, there are strong endogenous mutagenic processes required for the development of mature lymphocytes. This includes V(D)J recombination (Box 1) by RAG1/2 for antigen receptor diversity, AID (AICDA)-mediated class switch recombination (Box 1) and somatic hypermutation. Off-target activity of these cellular processes has been attributed to chromosomal translocations, such as translocation of the T cell receptor locus with its strong endogenous enhancers into close regulatory proximity to the oncogenes *LMO2*, *TAL1* and *TAL2* in T-ALL, and the AID-dependent *MYC*/*IGH* translocations in Burkitt's lymphoma (Marculescu et al., 2002; Robbiani et al., 2008). These endogenous mutagenic processes are a source of double-strand DNA breaks in developing lymphocytes, where off-target events are subjected to imperfect repair processes such as non-homologous end joining and homology directed repair (Helleday et al., 2014). Together, these processes can create lesions, including indels (Box 1), tandem duplications and translocations across the genome. Given RAG1/2 is allosterically activated upon binding to H3K4me3 (trimethylated lysine 4 of histone 3), a marker of active promoters, it is reasonable to postulate that genes that are co-expressed with RAG during cell development are at greater risk of off-target RAG endonuclease activity (Bettridge et al., 2017). There are also more generalised mutagenic processes at work in cancer genomes. Systematic analysis of somatic mutations in cancer genomes, including those of ALLs and AMLs, demonstrate that many mutations conform to mutational signatures associated with ageing and APOBEC-like cytidine deaminase activity (Box 1) (Alexandrov et al., 2013). This study also identified localised hypermutation termed kataegis (Box 1) in chronic lymphocytic leukaemia (CLL), B cell lymphoma and ALL genomes. Furthermore, the noncoding genome is subjected to higher mutation rates compared to its coding counterpart, although these mutations are under relatively weak selection pressures unless the mutations themselves confer a survival advantage (Weinhold et al., 2014). Given the many mutagenic processes at play, we should aim to open the search space for driver mutations in haematological malignancies beyond the exome to intergenic, intronic and promoter regions, in which such mutagenic events also occur.

Finally, there is merit in searching for noncoding mutations. A landmark discovery in 2013 showed that the promoter of *TERT*, which encodes the reverse transcriptase subunit of telomerase, can somatically acquire mutations leading to its overexpression in human melanoma (Huang et al., 2013). This finding was important in demonstrating that the noncoding genome itself can acquire driver mutations. Further work confirmed that these *TERT* promoter mutations recur in other malignancies, meaning similar variants were selected for during the development of multiple neoplasms (Horn et al., 2013; Vinagre et al., 2013). The mutations at the *TERT* promoter are excellent examples of noncoding driver mutations, as these lesions generate *de novo* consensus binding sites for ETS family TFs leading to consequential overexpression of *TERT*, telomere length maintenance through cell divisions, and thus continued cellular survival. Similar mutational hotspots have also been identified in noncoding regions of multiple cancer genomes. Examples include recurrent *FOXA1* promoter mutations, a known driver of hormone-receptor positive breast cancer, recurrent mutations in *cis*-regulatory elements that interact with the *ETV1* promoter in colorectal cancer and affect patient survival, and recurrent noncoding mutations in liver cancer (Fujimoto et al., 2016; Orlando et al., 2018; Rheinbay et al., 2017). These studies help to further rationalise the importance in searching for driver mutations in the noncoding genome.

## Architecture of a classical enhancer-promoter interaction

**Fig. 1.**
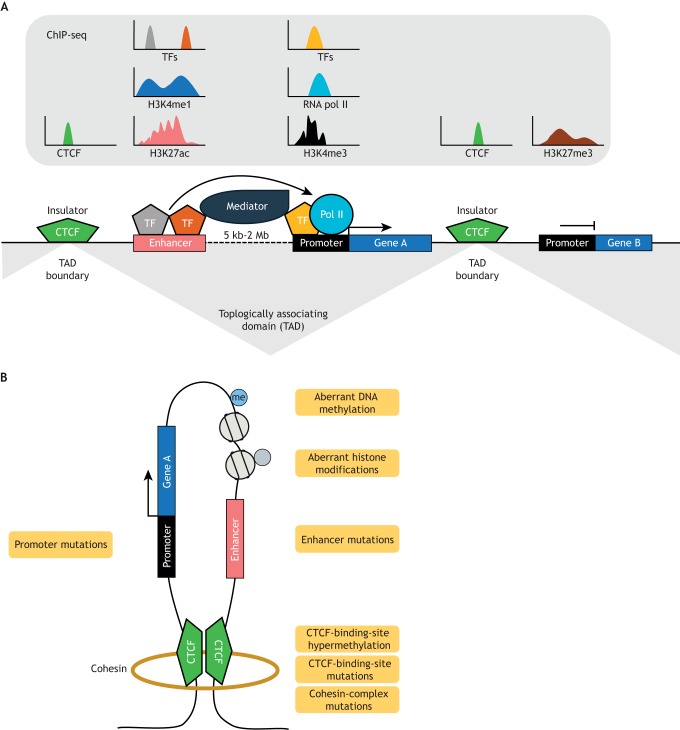
**Canonical enhancer to proximal promoter interaction.** (A) Transcription factors bind cognate sequences in the enhancer, which recruits cofactors, histone acetyltransferases and larger protein complexes such as Mediator. Loop formation between the enhancer and proximal promoter of the gene allows enhancer elements to interact with RNA pol II, followed by active transcription of the target genes. Enhancer-promoter interactions can be kept insulated from other genomic loci by the CTCF/cohesin complex, which closes this interaction into an insulated genomic neighbourhood, also known as a topologically associating domain. (B) An enhancer-promoter loop can be formed through somatically acquired mutations leading to aberrant gene regulation. These can arise from mutations that nucleate *de novo* regulatory elements such as promoters or enhancers, by mutations in noncoding sequences bound by CTCF or by mutations of the cohesin-complex members. Such mutation may yield detectable aberrations in DNA methylation and histone modifications.

Many of the noncoding mutations identified thus far in haematological malignancies appear to impinge on the pre-transcriptional regulation of genes. A number of regulatory noncoding elements interact before transcriptional initiation at a gene locus (Fig. 1B). This includes the promoter, which is located <1 kb upstream from the transcriptional start site (TSS; Box 1) of a gene and may include nearby proximal regulatory elements, and distal enhancers (Vernimmen and Bickmore, 2015). Whereas promoters effectively recruit the core transcriptional machinery, such as elongation factors and RNA pol II, it is enhancers that control spatiotemporal gene expression and tissue-specific gene expression programmes, often over vast genomic distances (Schoenfelder and Fraser, 2019). Importantly, enhancers can activate distal promoters independently of location and orientation relative to the target gene. Furthermore, enhancers are enriched with histone marks such as H3K27ac (histone 3 lysine 27 acetylation) and H3K4me1 (histone 3 lysine 4 monomethylation), and are bound by TFs and coactivators (Lambert et al., 2018; Medina-Rivera et al., 2018). Transmission of enhancer activity to RNA pol II at a gene promoter is achieved by the large multimeric protein complex called Mediator (Allen and Taatjes, 2015). Together, interactions with TFs and individual subunits of Mediator allow for stable association of these proteins with RNA pol II and the consequential formation of enhancer-promoter DNA loops. To ensure that the activity of enhancers is contained to specific target gene promoters, these enhancer-promoter loops are often contained within boundary elements, referred to as topologically associating domains (TADs), that are anchored by CCCTC-binding factor (CTCF) dimers and the cohesin (Box 1) complex (Hadjur et al., 2009; Hnisz et al., 2016a).

Enhancer-promoter interactions can be assembled *de novo* through somatic mutation of noncoding sequences (Fig. 1B). This can arise from activating promoter or enhancer mutations, which can result in aberrant activation of proto-oncogenes, and changes in histone modifications and/or DNA methylation. Furthermore, mutations of CTCF boundary elements, hypermethylation of CTCF-binding motifs, or cohesin-complex mutations, may disengage CTCF binding, leading to loss of enhancer insulation at TAD boundaries, which results in aberrant gene expression of neighbouring genes.

## Somatically acquired noncoding mutations and their mechanisms of action in haematological malignancies

### Noncoding mutations that nucleate *de novo* enhancers and promoters of oncogenes

Well-characterised examples of noncoding enhancer and promoter mutations have recently been identified in T-ALL genomes. In this cancer, translocations activating transcriptional regulators such as *LMO1*/*2*, *TAL1*/*2* and *TLX1*/*3*, together with the acquisition of activating mutations in the *NOTCH1* gene, are considered hallmarks of the disease (Liu et al., 2017; Pear et al., 1996; Weng et al., 2004). Both TAL and LMO TFs are expressed physiologically during early T cell differentiation and are then progressively silenced, unlike TLX TFs, which are not expressed in the T lineage (Dadi et al., 2012; Dik et al., 2005; Herblot et al., 2000). Together, dysregulation of the aforementioned genes leads to T cell differentiation arrest and rapid proliferation of malignant progenitors. Transcriptional analysis of primary patient samples showed that several oncogenic TFs, including *LMO2*, *TAL1* and *TLX1*, exhibited upregulated expression without an underlying cytogenetic lesion, leaving a subset of cases as genetically ‘unresolved’ (Ferrando and Look, 2003). In the case of TAL1-positive T-ALL, over half of the patient samples examined overexpress *TAL1*, many with monoallelic expression (Box 1) of *TAL1*, with no detectable cytogenetic aberration at the locus (Ferrando et al., 2004). This finding suggested that another mechanism (undiscovered at the time of this study) was activating the expression of *TAL1* in a subset of patients with T-ALL.

**Fig. 2.**
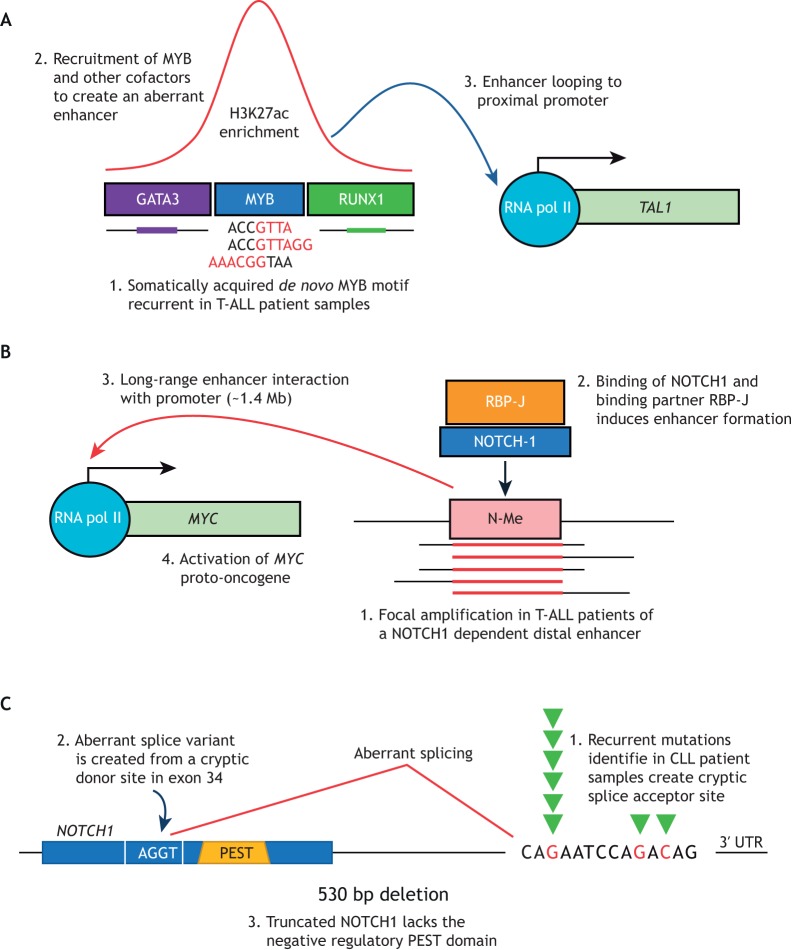
**Examples of noncoding mutations in haematological malignancies and mechanisms of action.** (A) Recurrent heterozygous somatic mutations of a noncoding element 5′ to *TAL1* creates a *de novo* binding site for the transcription factor MYB in T-ALL. Recruitment of MYB and additional cofactors leads to the formation of an aberrant enhancer, as seen by an enrichment of H3K27 acetylation. This enhancer is then able to interact with the proximal promoter of *TAL1* causing its monoallelic expression. (B) An endogenous NOTCH1-dependent enhancer is located 1.4 Mb away from the proximal promoter of *MYC* in T-ALL. Examination of primary patient samples demonstrated that this element is frequently and focally amplified in T-ALL, a cancer that frequently presents with mutations that constitutively activate NOTCH1. (C) Recurrent mutations in the 3′ UTR of *NOTCH1* were identified in CLL. These mutations lead to the formation of an aberrant splice acceptor site. A truncated splice variant is created between a cryptic splice donor site in the preceding exon and this mutant splice acceptor site. This allows for the formation of transcript that excises the negative regulatory PEST domain of NOTCH1, resulting in a more stable NOTCH1 protein.

Focusing on this subset of ‘unresolved’ TAL1-positive cases, our group identified a novel mechanism of oncogene activation, whereby a somatic mutation in a noncoding sequence ∼7 kb upstream from the *TAL1* TSS nucleated an aberrant transcriptional enhancer capable of driving TAL1 expression (Table 1) (Fig. 2A) (Mansour et al., 2014). In the study, somatically acquired heterozygous indels 2-18 bp in length introduced binding motifs for the TF MYB at this precise noncoding site. These noncoding mutations were found in 5.5% of unselected paediatric primary T-ALL patient samples, making this region a mutation hotspot.

**Table 1.**
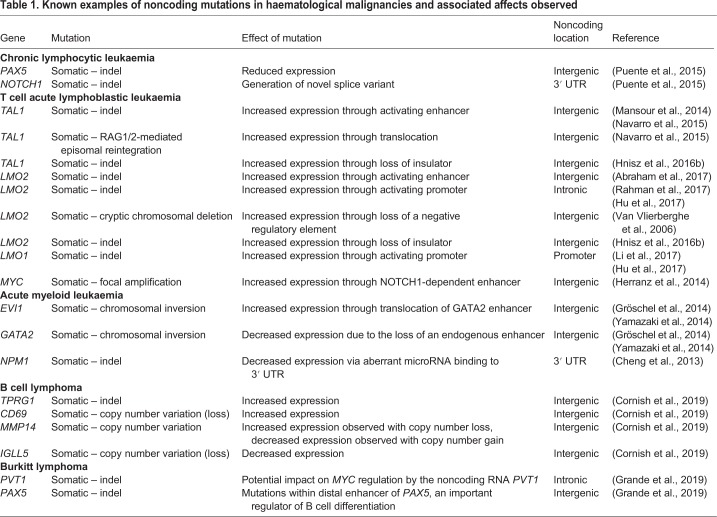
**Known examples of noncoding mutations in haematological malignancies and associated affects observed**

The fact that these mutations created MYB binding sites was a significant observation. MYB is a master TF in T-ALL and capable of recruiting the TAL/LMO complex assembled from multiple transcriptional activators, including GATA3, HEB (TCF12), RUNX1, the histone acetyltransferase CBP (CREBBP), and TAL1 itself. Biochemical assays have shown that direct binding between MYB and CBP through its KIX domain is required for MYB-dependent transcriptional activation (Zor et al., 2004). Indeed, recruitment of the TAL/LMO complex to this noncoding site upstream of *TAL1* was validated using ChIP-seq (Box 1) in the Jurkat T-ALL cell line and the locus was enriched for H3K27 acetylation, a marker of an active enhancer facilitated by intrinsic CBP histone acetyltransferase activity. This led to robust expression of *TAL1* from the mutant allele, and no expression from the wild-type allele, explaining the monoallelic expression of *TAL1* described in the Jurkat cell line many years before (Leroy-Viard et al., 1994). It is also congruent that a TF such as MYB, which relies on explicit recognition and binding of a DNA sequence, would be able to exploit a somatic mutation in the noncoding genome. All the mutations identified in primary patient samples either matched or were similar to the cognate MYB binding sequence, allowing for MYB-dependent enhancer formation. Furthermore, CRISPR/Cas9-mediated excision of the mutation in the Jurkat T-ALL cell line led to full abrogation of *TAL1* expression (Mansour et al., 2014).

Navarro et al. also independently reported *cis*-acting lesions in the same noncoding locus upstream of *TAL1* in T-ALL, leading to diminished H3K27 trimethylation (Table 1) (Navarro et al., 2015). This study also reported the first case of RAG1/2-mediated episomal reintegration in the same noncoding locus. This event integrated a ∼370 kb section of chromosome 7 flanked by recombination signal sequences leading to aberrant *TAL1* expression, demonstrating that this specific noncoding site is able to transcriptionally activate *TAL1* through differing genetic lesions.

Similar to the TAL1-positive cases, a proportion of T-ALL samples also overexpress *LMO2* without a known cytogenetic lesion. Using aberrant ChIP-seq peaks as a discovery tool, our group identified a mutation hotspot within intron 1 of *LMO2*, leading to the formation of an aberrant promoter (Table 1) (Rahman et al., 2017). These somatically acquired *cis*-acting heterozygous mutations create high-confidence *de novo* TF binding motifs, not only for MYB, but also for ETS1 and RUNX1. These somatic *LMO2* mutations were identified in 3.7% of paediatric and 5.5% of adult T-ALL patient samples and in the PF-382 and DU.528 T-ALL cell lines, suggesting that these mutations were indeed driver events. Analysis of 5′ capped RNA transcripts in the mutant cell lines showed that *LMO2* transcripts skipped exon 1 and instead started transcription from exon 2, confirming that these intronic mutations generate an aberrant *cis*-acting promoter. Interestingly, a nascent MYB motif already exists in this site, with several of the mutations introducing a second MYB site 10 or 20 bp away, suggesting MYB may bind to this mutant locus as a dimer, separated by one or two helical coils of DNA. Indeed, CRISPR/Cas9-mediated deletion that disrupts this spacing led to an almost total loss of *LMO2* expression (Rahman et al., 2017).

Again using ChIP-seq as the discovery tool, somatically acquired noncoding mutations in the *LMO1* oncogene were also identified in 2% of paediatric T-ALL samples and in the Jurkat T-ALL cell line (Table 1) (Li et al., 2017). Li et al. described a single C-to-T nucleotide transition 4 kb upstream of *LMO1* that created a *de novo* MYB binding site, leading to high levels of *LMO1* expression, although it is unclear whether the mutations create an enhancer or a promoter. Further analysis of this mutation by the authors demonstrated that it conforms to an APOBEC-like cytidine deaminase mutation signature, characterised by TCN-to-TTN changes. This mutational signature has been observed across multiple cancer types, beyond those of lymphoid origin, suggesting that somatically acquired oncogenic promoter or enhancer mutations may be found in other cellular lineages (Alexandrov et al., 2013).

In an independent study, Hu et al. used whole-genome sequencing (WGS) of 31 paediatric T-ALL patients and matched germline samples to identify the previously discussed somatically acquired noncoding variants at the *TAL1* and *LMO2* loci, but also the *LMO1* promoter mutation (Table 1) (Hu et al., 2017). The authors also transduced the Jurkat T-ALL cell line with a doxycycline-inducible CRISPR/Cas9 system targeting the enhancer mutation and injected these engineered cells into immunocompromised mice to evaluate the effects of the *LMO1* enhancer mutation *in vivo*. Importantly, mice on a doxycycline-supplemented diet, in which the activated CRISPR/Cas9 reagents excised the *LMO1* enhancer mutation and thus suppressed LMO1 overexpression, exhibited lower leukaemic engraftment at week 4 and improved survival compared to mice on a regular diet, providing clear evidence that this enhancer variant is important for the pathogenesis of this leukaemia.

### Genetic lesions that alter the function of endogenous enhancers

*Cis*-acting enhancers can regulate genes across vast genomic distances. An excellent example of this is the NOTCH1-bound *MYC* enhancer (N-Me) in T-ALL, which is situated +1.47 Mb relative to the *MYC* TSS (Table 1) (Fig. 2B) (Herranz et al., 2014). Unlike the previous examples, this *cis*-acting enhancer is not acquired through mutation, but is a highly conserved endogenous regulatory sequence with a critical role in T cell development. The authors demonstrated that NOTCH1 binds to this *cis*-acting element along with its binding partner RBPJ, leading to transcriptional activation of *MYC.* Analysis of primary T-ALL patient samples identified focal duplications of chromosome 8q24, which includes the N-Me locus, in 5% of cases, suggesting these leukaemias amplify this locus to enhance MYC expression. The authors also determined that this enhancer is essential for normal thymic development. Deletion of N-Me reduced thymic cellularity and mature T cell counts, but made knockout mice resistant to NOTCH1-induced T-ALL. Further analysis of this regulatory element has identified the role for GATA3-mediated nucleosomal eviction in its activation (Belver et al., 2019)

The examples described thus far illustrate the gain-of-function capability of noncoding aberrations in driving oncogene expression. In a study of CLL, the converse was shown to be true – noncoding lesions leading to the inactivation of a tumour suppressor gene. Here, recurrent indels inactivate the B cell developmental enhancer of *PAX5*, leading to PAX5 haplosufficiency (Table 1) (Puente et al., 2015). Importantly, the authors functionally validated the enhancer using CRISPR/Cas9 genome editing in which deletions of the putative enhancer in a lymphoblastoid B cell line led to a 40% reduction in *PAX5* expression. A similar mechanism has also been described in the inherited MonoMAC syndrome (Box 1), in which mutations inactivate a *GATA2* intronic enhancer leading to GATA2 haplosufficiency (Hsu et al., 2013).

Genetic lesions that alter the function of endogenous enhancers have also been characterised in AML. Gröschel et al. showed that recurrent inv(3)(q21q26.2) or t(3;3)(q21;q26.2) [inv(3)/t(3;3)] rearrangements, which lead to aberrant expression of the proto-oncogene *EVI1* in AML, are caused by the translocation of an endogenous enhancer which usually regulates *GATA2* (Table 1) (Gröschel et al., 2014). These genetic lesions translocate an 18 kb noncoding sequence to the *EVI1* (*MECOM*) gene locus. ChIP-seq analysis of the inv(3) MOLM-1 AML cell line defined a putative enhancer element within this translocated 18 kb noncoding sequence owing to its significant enrichment for the p300 transcriptional coactivator (EP300) and for the H3K27ac, H3K4me3 and H3K4me1 histone marks. Interestingly, the authors also show that loss of the enhancer from its usual resident position in the genome leads to *GATA2* haploinsufficiency and to its monoallelic expression from the unaffected allele. Cloning of this enhancer sequence into a reporter assay resulted in strong reporter gene induction within the inv(3) myeloid cell lines MUTZ-3 and MOLM-1, but not in non-myeloid cell lines. This suggested that this enhancer required myeloid-specific transcriptional components to activate. Excision of this p300-bound translocated enhancer with TALE nucleases silenced *EVI1* expression and stalled cellular growth in the inv(3) AML cell line, further demonstrating the driver nature of this genetic aberration. This finding was confirmed in a complimentary *in vivo* study by Yamazaki et al., in which a bacterial artificial chromosome encoding the inversion generated a transplantable leukaemia in mice (Table 1) (Yamazaki et al., 2014). The inv(3)/t(3;3) offers a good example of enhancer hijacking, and shows how a single genetic lesion can lead to activation of an oncogene and inactivation of a tumour suppressor in a single ‘hit’.

### Noncoding variants that lead to aberrant splicing

Alternative splicing facilitates greater transcriptional diversity from a single precursor messenger RNA under differing cellular contexts. Most human genes (94%) can generate different mRNA isoforms, which can ultimately be translated into different proteins (Gerstein et al., 2014; Pan et al., 2008). Data from TCGA has shown that cancer genomes can acquire somatic mutations that alter normal splicing mechanisms, including intron retention, which is thought to inactivate tumour suppressors (Jung et al., 2015). By analysing genome-wide patterns of RNA splicing in AML genomes, Dvinge and Bradley identified that the extent of intron retention correlated with mutations in *RUNX1*, *IDH1* and *IDH2* relative to wild-type AML samples. The authors postulate that differential methylation arising from *IDH1*/*2* mutations may have an impact on RNA splicing through a crosstalk mechanism, although they did not experimentally investigate this (Dvinge and Bradley, 2015). TCGA data have also shown that AMLs can acquire somatic mutations within the noncoding sequences that demarcate the two positions that should be spliced together, also known as the 5′ splice donor and the 3′ splice acceptor sites (Kandoth et al., 2013).

By interrogating CLL genomes by WGS, Puente et al. identified recurrent mutations in the noncoding 3′ untranslated region (UTR) of *NOTCH1* (Table 1; Fig. 2C) (Puente et al., 2015). RNA-seq examination of the mutated cancers confirmed the presence of aberrant splicing of *NOTCH1*, where the final exon was spliced into a newly formed splice acceptor site in the 3′ UTR of *NOTCH1*. As a result of this, 158 coding bases were deleted, including those encoding the negative regulatory PEST domain of the mature NOTCH1 protein. Loss of this domain interferes with the ubiquitination-mediated degradation of NOTCH1, giving rise to increased levels of activated NOTCH1. Indeed, immunohistochemistry confirmed the presence NOTCH1 in the nuclei of mutant CLL cells, and data from patients with the 3′ UTR *NOTCH1* mutations suggested that this mutation was concomitant with adverse prognosis.

### Genetic lesions that disrupt negative regulatory noncoding sequences

In addition to enhancers, the noncoding genome is also rich with negative regulatory sequences, or insulators. These block the activity of enhancers, and are usually positioned between the enhancer of a gene and its proximal promoter. Negative regulatory elements have been identified upstream of the *LMO2* gene. In a study by Hammond et al., the authors set out to understand the contribution of upstream noncoding sequences on the regulation of *LMO2* expression (Hammond et al., 2005). First, they cloned 3190 bp 5′ to the TSS into luciferase reporters and tested reporter activity in different cell lines. In erythroid cells, where *LMO2* is constitutively expressed, expression of the reporter gene was maintained, however the Jurkat T-ALL cell line, where *LMO2* is not active, had significantly reduced reporter activity. This suggested the presence of a tissue-specific negative regulatory element within the noncoding sequence. Building on this research, Van Vlierberghe et al. identified a recurrent cryptic deletion del(11)(p12p13) in T-ALL using the array-comparative genome hybridisation (array-CGH) technique (Table 1) (Van Vlierberghe et al., 2006). This cryptic deletion led to the loss of the aforementioned negative regulatory region of *LMO2*, leading to aberrant activation of the proximal promoter and *LMO2* expression. A follow up study successfully improved on the detection method by using a multiplex ligations probe assay to identify additional monoallelic LMO2 expressors with deletions of the negative regulatory element (Van Vlierberghe et al., 2008).

Higher-order chromatin conformation can regulate genes by dynamically altering the interactions between gene promoters and regulatory elements. These are often held together in DNA loops, where the loop itself is closed by the binding of two CTCFs, creating an insulated neighbourhood. These loops can further be organised in larger TADs (Dixon et al., 2012; Dowen et al., 2014). This led Hnisz et al. to postulate that mutations could affect the boundary elements between insulated neighbourhoods, which in turn may lead to the deregulated expression of proto-oncogenes (Table 1) (Hnisz et al., 2016b). The authors mapped the Jurkat T-ALL cell line genome with chromatin interaction analysis by paired-end tag sequencing (ChIA-PET; Box 1). This technique allowed for the identification of CTCF-CTCF looping interactions across the genome. Notably, the authors showed that *TAL1* is located in its own insulated neighbourhood in Jurkat cells, with one CTCF site found in the middle of a neighbouring gene called *STIL*, and the other located in a noncoding region 3′ of *TAL1*. Interestingly, a subset of primary T-ALL patient samples appeared to have genomic deletions of the CTCF site within *STIL*, which in effect destroys the insulation boundary of the neighbourhood. This now meant that *TAL1* could be regulated by distal enhancers, which it would usually be insulated from, leading to its overexpression. Similar mutations were also found in patient samples at the *LMO2* locus, although it is unclear whether these also deleted the previously discussed negative regulatory element of *LMO2* (Van Vlierberghe et al., 2008). CRISPR/Cas9-mediated deletion of CTCF-associated boundary sites in HEK293T cells led to an almost 2-fold increase in both *TAL1* and *LMO2* expression, demonstrating that loss of the insulated neighbourhood in an unrelated cell line can upregulate the expression of these proto-oncogenes.

Although recurrent mutations disrupting CTCF motifs themselves have been described in solid tumours (Katainen et al., 2015), to our knowledge, such mutations have not been described in haematological malignancies. However, it is notable that CTCF binding to DNA is sensitive to DNA methylation (Bell and Felsenfeld, 2000; Hark et al., 2000). In gliomas, Flavahan et al. describe how gain-of-function IDH family gene mutations leads to the production of an onco-metabolite called 2-hydroxygluterate, which interferes with the function of TET family 5′-methylcytosine hydroxylases (Flavahan et al., 2016). This ultimately leads to increased DNA methylation, which, when occurring at CTCF sites, impairs CTCF binding. The authors demonstrate that methylation of a CTCF site downstream of *FIP1L1* leads to loss of insulation and upregulation of the neighbouring oncogene *PDGFRA.* Given the prevalence of *IDH1*/*2* and *TET2* mutations in AML, it is tempting to speculate that similar mechanisms are at play in this disease (Cancer Genome Atlas Research Network et al., 2013a; Marcucci et al., 2010; Papaemmanuil et al., 2016). Furthermore, many TFs, including MYB, have recently been shown to have altered affinity for methylated DNA, and it is therefore likely that DNA methylation plays an important role in the transcriptional dysregulation seen in many haematopoietic malignancies. (Yin et al., 2017).

## Challenges and potential methods to identify novel functional noncoding mutations

### Challenges

The identification and characterisation of noncoding mutations in haematological malignancies is fraught with challenges. The principal problem is to separate driver mutations from passenger mutations. Intrinsic to this issue is that variants in the noncoding genome do not alter amino acids, which means that immediate functional characterisation of a variant is not possible. Furthermore, passenger mutations are acquired over the course of an average human lifespan, which could occur before or after the acquisition of a driver mutation. Martincorena and Campbell discuss this in their review and also state the importance of sequencing normal tissues alongside malignant ones to distinguish passenger mutations from rare germline variants (Martincorena and Campbell, 2015). An important challenge is to isolate mutations that are positively selected for during the development of the malignancy, and under this circumstance, mutational recurrence and allele frequency across primary patient samples may provide a clue.

Given the vastness of the noncoding genome, another challenge is to know where to look for driver mutations. In much of the noncoding genome, variations will have no observable phenotypic affect, therefore it is important to restrict search space to sequences that may have regulatory potential on other genes. To this end, acquisition of datasets that provide insights into accessible regions of chromatin, and the position of active enhancers or promoters may be valuable. Without further experimental interrogation, it can still be difficult to connect specific enhancers to the target genes that these elements regulate. It is therefore also important to establish causality with a possible phenotypic outcome. Genome engineering techniques such as CRISPR/Cas9 have been crucial in this regard, as they allow for the reversion of genetic lesions back to wild type, or to knock in mutations where they were not present before and then assess the phenotype.

### Proposed methods to identify functional noncoding variants

A number of differing methods have been employed to identify functional noncoding variants. Some studies, including those from our own research group, have successfully examined biological samples that exhibit monoallelic expression of proto-oncogenes without underlying cytogenetic lesions at the locus (Li et al., 2017; Mansour et al., 2014; Rahman et al., 2017). In these studies, integrated datasets have been helpful where ChIP-seq for H3K27 acetylation and the TF MYB has limited search space for noncoding sequences with regulatory potential, which have then ultimately harboured recurrent somatically acquired mutations in primary patient samples. Monoallelic expression can be identified by RNA-seq, albeit with a number of challenges. Some notable examples being that genes imprinted as part of mammalian development will be identified, and such genes may not harbour *cis*-acting lesions or be relevant to the disease state. Furthermore, accurate calling of monoallelic expression relies on an awareness of heterozygous SNP positions in exons or UTRs to appropriately distinguish expression between two alleles. Genes that have no heterozygous SNPs cannot be assessed for allelic expression using this method, limiting its utility as a truly genome-wide approach.

Chromatin accessibility can indicate the presence of active enhancers or promoters. These can be determined through multiple techniques, including ChIP-seq, assay for transposase-accessible chromatin sequencing (ATAC-seq), and DNase-seq (Box 1) (Boyle et al., 2008; Buenrostro et al., 2013, 2015). Abraham et al. developed a novel bioinformatic pipeline to examine H3K27 acetylation ChIP-seq reads for indels (Table 1) (Abraham et al., 2017). H3K27ac limits the search space to only ∼2% of the genome, and is particularly suited for discovery of gain-of-function mutations. This pipeline effectively uncovered a recurrent mutation hotspot 40 kb upstream from *LMO2* that forms an aberrant enhancer to drive *LMO2* expression in T-ALL.

Integration of WGS with ATAC-seq can also identify cancer-relevant mutations in the noncoding genome (Corces et al., 2018). In this study, mutations were identified in ATAC-seq reads with variant allele frequencies (VAF) >80%, even though the same variant was identified as heterozygous with a 50% VAF by WGS reads. This discrepancy suggested that the mutant allele was more accessible to transposase and the site of a novel *cis*-regulatory element. This approach successfully characterised a noncoding enhancer mutation creating a predicted *de novo* binding site for the TF NKX2-8, leading to upregulation of *FGD4*, an important regulator of the actin cytoskeleton in bladder cancer. In a recent study, WGS from 117 patients with B cell lymphoma was coupled with promoter capture Hi-C (Box 1) data from naïve B cells to define *cis*-regulatory elements (Table 1) (Cornish et al., 2019). Using this method, the authors identified copy number variations and single nucleotide variants at *cis*-regulatory elements that affect the expression of *MMP14* and *IGLL5*, where low expression is believed to be associated with poor prognosis. The authors also identified aberrations that affected the expression of *CD69* and *TPRG1*, where increased expression has been previously implicated in lymphoma oncogenesis.

**Fig. 3.**
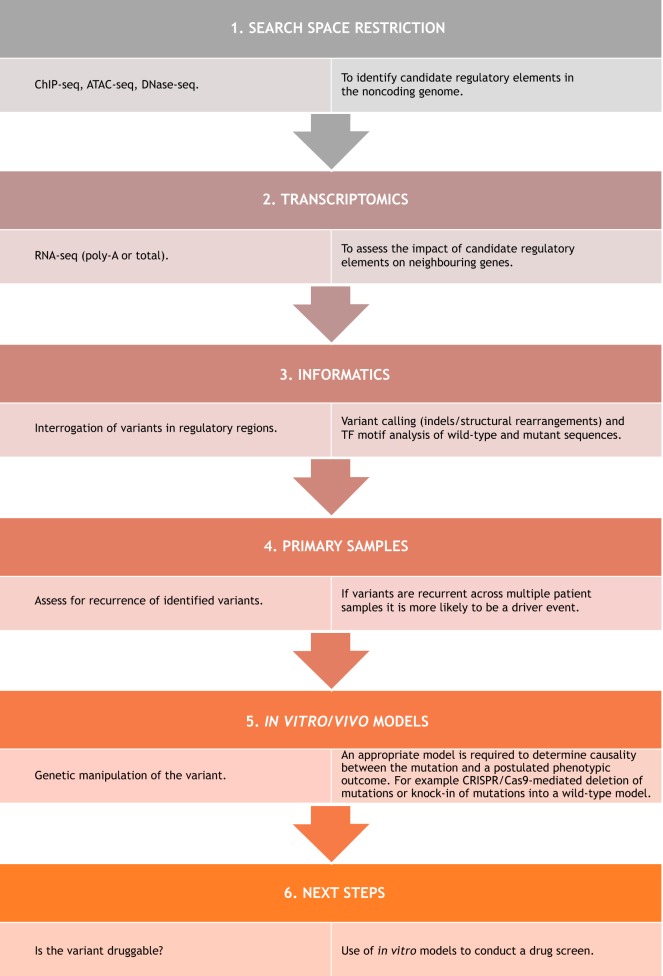
**Framework for identifying novel noncoding mutations.** Proposed research framework for the successful identification and functional characterisation of noncoding variants in human malignancies.

By examining the existing literature, it is possible to generate a framework to identify noncoding mutations (Fig. 3). In almost all studies, the search space for novel mutations was restricted by exploring a gene of interest previously implicated in disease pathology, or by identifying regulatory elements in the noncoding genome. Studies also had access to transcriptomic data to assess the impact of newly identified regulatory regions on candidate genes. Given the complexity of the data from sequencing runs, robust bioinformatic analysis is crucial to identify novel indels or to subject candidate noncoding regions to motif analysis (Hume et al., 2015; Khan et al., 2018; Matys et al., 2003). This may help identify *de novo* TF binding motifs, which itself may provide insight into the mechanism of action. If a variant is a true oncogenic noncoding mutation, it should be recurrent, and so access to primary patient-derived samples to validate the presence of variants is important, and such variants should be absent from germline controls, or absent from remission samples if indeed they are somatically acquired. Finally, detailed mechanistic insights have been achieved by examining noncoding mutations in suitable cellular or organismal models. These variants are best explored within their endogenous loci, hence detailed phenotypic assessment should be attempted following CRISPR/Cas9-mediated excision of the mutations in models which already harbour the mutation, or knock-in of the mutations into a wild-type locus.

## Potential therapeutic interventions

Somatically acquired noncoding mutations identified thus far in haematological malignancies have common molecular features. As described in this Review, these variants create stable and potent enhancers with massive enrichment of H3K27 acetylation, orders of magnitude above that of other endogenous enhancers. Some researchers have postulated that these enhancers are in effect ‘super-enhancers’, locus-control regions or just ‘strong’ enhancers (Pott and Lieb, 2015). Although there is continued debate regarding semantics, there is growing consensus that these enhancer complexes can be disassembled through therapeutic intervention. Stable enhancers are usually occupied by RNA pol II, Mediator, cohesin, p300, CBP and the bromodomain and extraterminal (BET) protein BRD4 (Hnisz et al., 2013). BET proteins such as BRD4 detect histone acetylation at specific genomic loci and TF binding events, which then further recruits the Mediator complex and RNA pol II elongation factors (Shi and Vakoc, 2014).

Lovén et al. examined the effects of the BET inhibitor JQ1 in multiple myeloma (Lovén et al., 2013). In the study, the authors observed that treatment with JQ1 depletes BRD4 specifically at ‘super-enhancers’ that are massively enriched with MED1 and BRD4. This included the selective inhibition of the enhancers that regulate the *MYC* oncogene. This was a particularly significant finding as it suggested there could be a therapeutic window to selectively inhibit the strongest enhancers, which in turn regulate the strongest oncogenes, in this malignancy. Further success with JQ1 was also described by Gröschel et al., where treatment with the compound led to significantly reduced *EVI1* expression and growth arrest of inv(3) AML cells, but no sensitivity in cells that have no rearrangement at that locus and yet overexpress *EVI1* (Gröschel et al., 2014). The authors postulate that this is because there is an active ‘super-enhancer’ driving *EVI1* expression within inv(3) AML cells. Recent evidence suggests that BET degraders may also be an effective way to stop aberrant enhancer activity (Winter et al., 2017). Unlike BET inhibitors such as JQ1, which may only be effective in a subset of massively enriched enhancer regions, the authors state that a BET degrader called dBET6 leads to a total collapse of global transcriptional elongation, at a similar magnitude of CDK9 inhibition. By treating T-ALL cells with dBET6, the authors demonstrate efficacy of the degrader at lower concentrations compared to JQ1, and increased survival of T-ALL-engrafted mice treated with the degrader, compared to mice treated with the inhibitor. Furthermore, inhibitors of the proteins that regulate C-terminal domain phosphorylation of RNA pol II have been shown to effectively downregulate the expression of genes that are regulated by enhancers. This includes the CDK7 inhibitor THZ1, which leads to potent suppression of the *TAL1* enhancer in Jurkat T-ALL cells, raising the possibility that targeting noncoding drivers of oncogenes may offer an exploitable therapeutic window in cases where oncogenes are aberrantly expressed but do not carry an obvious cytogenetic change (Kwiatkowski et al., 2014).

## Future outlook

Recent advances have made it clear that the noncoding genome is not only acquiring passenger mutations, but also driver mutations that cause the dysregulated expression of proto-oncogenes in haematological malignancies. It is probable that such mutations exist in other malignancies, but have remained under-reported owing to the challenges faced with initial discovery and subsequent functional characterisation. As we continue into the genomic age, researchers are generating vast integrated datasets that include WGS, which can readily identify mutations, RNA-seq for the quantification of gene expression, and ChIP/ATAC/DNase-seq, all of which are highly effective at mapping gene regulatory elements. This provides an opportunity to not only discover novel noncoding mutations, but to assess these mutations for phenotypic outcomes, and to establish cause and effect with the assistance of genetic perturbation and gene editing technologies that have become ubiquitous in translational research. Identifying such mutations has several potential implications; they may have prognostic relevance, large indels may be amenable to minimal residual disease analysis, they can highlight novel oncogenes that are potentially druggable, and they can provide important insights into the optimal DNA syntax required for nucleation of multiprotein TF complexes. The factors that interplay to orchestrate transcriptional regulation in normal development, and transcriptional dysregulation in cancer, are highly complex. It may be that noncoding mutations selected for during tumorigenesis will prove to be ‘experiments of nature’, the Rosetta stone that helps us understand the language of the noncoding genome.
